# Chiral spin ordering of electron gas in solids with broken time reversal symmetry

**DOI:** 10.1038/s41598-019-47274-6

**Published:** 2019-07-25

**Authors:** K. S. Denisov, I. V. Rozhansky, N. S. Averkiev, E. Lähderanta

**Affiliations:** 1 Ioffe Institute, St.Petersburg, 194021 Russia; 2 Lappeenranta-Lahti University of Technology, FI-53851 Lappeenranta, Finland

**Keywords:** Spintronics, Magnetic properties and materials, Surfaces, interfaces and thin films, Two-dimensional materials

## Abstract

In this work we manifest that an electrostatic disorder in conducting systems with broken time reversal symmetry universally leads to a chiral ordering of the electron gas giving rise to skyrmion-like textures in spatial distribution of the electron spin density. We describe a microscopic mechanism underlying the formation of the equilibrium chiral spin textures in two-dimensional systems with spin-orbit interaction and exchange spin splitting. We have obtained analytical expressions for spin-density response functions and have analyzed both local and non-local spin response to electrostatic perturbations for systems with parabolic-like and Dirac electron spectra. With the proposed theory we come up with a concept of controlling spin chirality by electrical means.

## Introduction

The concept of spin chirality constitutes a substantial part of modern condensed matter physics. It is widely applied to strongly correlated electron systems^1–4^ when interpreting the fractional statistics^5–7^, or chiral spin liquids^8–11^ in terms of an effective gauge field. Remarkably, a finite spin chirality induces a gauge invariant magnetic flux, which is an experimentally observable quantity^1^. It was shown that the chirality driven magnetic field affects electron transport in the very same way as the ordinary magnetic field does^12,13^ leading to the Hall response, the phenomenon currently referred as the topological Hall effect^14–17^. Naturally, to get an experimental access to the variety of spin chirality driven phenomena an efficient tool for creating a chiral spin order is needed. One way towards this goal is to focus on materials possessing exotic spin textures, such as magnetic skyrmions^18–21^, or merons^22^. Magnetic skyrmion is a topologically nontrivial configuration of the magnetization stabilized by a spin-orbit interaction in systems with broken inversion symmetry^23,24^; along with the fact that it triggers a number of peculiar effects, such as the skyrmion Hall effect^25^, this quasiparticle attracts a great interest due to possible applications in the race-track memory devices^26,27^. Still, exploring the physical mechanisms behind the emergence of spin chirality in solids remains challenging and is of high fundamental interest.

In this paper we show that in systems with broken time reversal symmetry (${\mathscr{T}}$-symmetry) a chiral spin order of electron gas is universally induced by an electrostatic disorder, which is an inherent property of any real solid. We argue that numerous crystal imperfections, such as residue impurities or surface defects appear to be a source of local chiral spin ordering of electrons. This effect is more pronounced for an electron gas with stronger spin-orbit interaction (SOI). Naturally, various magnetic systems such as magnetic topological insulators (TI)^28–31^, Rashba magnetic layers^32–35^ or dilute magnetic semiconductors (DMS)^36–41^ are in fact flooded by chiral spin textures pinned to structural defects. This effect opens up a novel concept of an experimental research of spin chirality driven phenomena.

## General Theory

In our work we focus on two-dimensional degenerate electron gas (2DEG). We introduce an effective ‘magnetic field’ acting on an electron spin:

1 $${{\boldsymbol{B}}}_{k}=(\lambda k\,\cos (\chi {\phi }_{k}+\gamma ),\lambda k\,\sin (\chi {\phi }_{k}+\gamma ),h),$$

where ***k*** = (*k*,*φ*_*k*_) is a 2D momentum with magnitude *k* and polar angle *φ*_*k*_. The parameter *h* > 0 describes the out-of-plane component leading to the carrier spin splitting at *k* = 0, it is thus responsible for the violation of ${\mathscr{T}}$-symmetry. The in-plane components of ***B***_*k*_ represent linear in *k* terms due to SOI, *λ* is the SOI coupling constant. The SOI parameters *χ* = ±1 (helicity) and *γ* (vorticity) cover different types of the SOI interaction.

Let us further assume an electrostatic disorder due to various defects present in the system. When ${\mathscr{T}}$-symmetry is broken the spatial distribution of the equilibrium electron spin density follows the inhomogeneity of the electrostatic potential *V*(***r***) (the notation ***r*** = (*x*,*y*) stands for 2D radius vector, we do not take into account variation of the potential along *z*-axis perpendicular to the motion plane). Indeed, at *h* ≠ 0 there is a nonzero electron spin polarization directed perpendicular to the motion plane. A local variation of the potential magnitude *V*(***r***) leads to a spatial redistribution of electrons and, hence, to the change of both 2DEG charge density and spin density *δS*_*z*_ component along *z*. When SOI is present (*λ* ≠ 0) the in-plane components of the spin density *δS*_*x*,*y*_ appear as well, so the induced spin response *δ****S*** acquires a chiral spatial pattern forming skyrmion-like spin textures.

**Figure 1:**
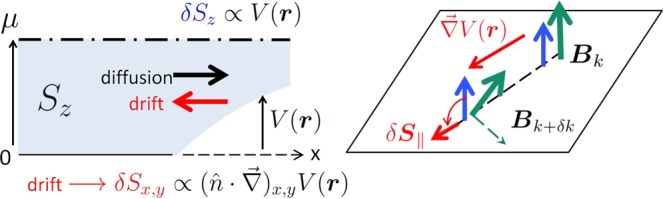
The physical picture behind the emergence of an equilibrium chiral spin pattern of the electron gas. The in-plane spin arises from the precession due to drift electron flow.

Let us notice that the mechanics behind the appearance of *δS*_*x*,*y*_ in response to an electrostatic potential has a peculiar character, and it differs from that for *δS*_*z*_. Since there is no net in-plane spin polarization at a spatially uniform electrostatic potential, *δS*_*x*,*y*_ appears only due to its gradient. One can consider the following quasiclassical picture, see Fig. 1. An electron with initial momentum ***k*** and spin ***S***_*k*_ co-aligned with the direction of ***B***_*k*_ moves along a certain trajectory. Due to the electrostatic potential gradient $\overrightarrow{\nabla }V({\boldsymbol{r}})$ the carrier momentum is changed ***k*** + *δ****k***, thus changing the tilt of ***B***_*k*+*δk*_, which in-plane components are coupled with momentum. This process triggers the precession of the electron spin around the new direction of the effective magnetic field creating an excessive in-plane spin density. In the thermodynamic equilibrium there is no net current as the drift and diffusion electron flows are compensated everywhere. However, when the time-reversal symmetry is broken the two flows have different effect on an electron spin. Indeed, while the diffusion flow does not affect the in-plane spin components, the drift flow associated with the change of the electron momentum leads to the emergence of an in-plane spin according to the arguments given above. This difference between drift and diffusion flows results in the accumulation of 2DEG spin density *δS*_*x*,*y*_ in the equilibrium.

The emergence of the spin textures due to spatial variation of the electrostatic potential is described by static spin-density response functions:

2 $${ {\mathcal F} }_{\alpha }({\boldsymbol{q}})=\sum _{k,s,s^{\prime} }\,\langle {u}_{k}^{s}|{\hat{S}}_{\alpha }|{u}_{k+q}^{s^{\prime} }\rangle \langle {u}_{k+q}^{s^{\prime} }|{u}_{k}^{s}\rangle \frac{{f}_{k}^{s}-{f}_{k+q}^{s^{\prime} }}{{\varepsilon }_{k}^{s}-{\varepsilon }_{k+q}^{s^{\prime} }+i0},$$

where ${\hat{S}}_{\alpha }$ is a spin projection operator for *α* = (*x*, *y*, *z*) axis, index *s* = ± denotes two electron subbands, ${\varepsilon }_{k}^{s}$ and $|{u}_{k}^{s}\rangle$ are the energy and the Bloch amplitude of an electron in the state (***k***, *s*), ${f}_{k}^{s}$ is the equilibrium distribution function. Using the functions ${ {\mathcal F} }_{\alpha }({\boldsymbol{q}})$ one can analyze the spin density *δ****S***(***r***) emerging in 2DEG in the vicinity of a doping center or a defect with a potential *V*(***r***):

3 $$\delta {{\boldsymbol{S}}}_{\alpha }({\boldsymbol{r}})=\int \,\frac{d{\boldsymbol{q}}}{{\mathrm{(2}\pi )}^{2}}{e}^{i{\boldsymbol{qr}}}{ {\mathcal F} }_{\alpha }({\boldsymbol{q}})V({\boldsymbol{q}}),$$

where *V*(***q***) is the Fourier transform of *V*(***r***), the electron-electron interaction is neglected. In particular, the functions ${ {\mathcal F} }_{\alpha }({\boldsymbol{q}})$ allow us to identify whether the spin response is local or extends beyond the localization radius of the potential due to the wave properties of 2DEG.

Let us point out a few general features of the spin response. As has been mentioned above, *z*-component of the 2DEG spin density is analogous to the 2DEG charge density, so if there is no spatial dispersion *δS*_*z*_ locally couples with the potential $\delta {S}_{z}({\boldsymbol{r}})={\varkappa }_{z}V({\boldsymbol{r}})$. On the contrary, the in-plane spin components driven by the precession mechanism illustrated by Fig. 1 are induced by the gradient of *V*(***r***). In the case of a local response this coupling takes the form $\delta {S}_{x,y}={\varkappa }_{\parallel }{(\hat{n}\cdot \overrightarrow{\nabla })}_{x,y}V({\boldsymbol{r}})$, where $\hat{n}$ is a rotation matrix determined by SOI type (see Eq. 4). The coefficients ${\varkappa }_{z,\parallel }$ are determined by a carrier spectrum. Since the Fourier transform of $\overrightarrow{\nabla }V({\boldsymbol{r}})$ is *i****q****V*(***q***), we conclude that ${ {\mathcal F} }_{x,y}({\boldsymbol{q}})$ are purely imaginary, we express them as

4 $${ {\mathcal F} }_{x,y}({\boldsymbol{q}})=i{(\hat{n}{{\boldsymbol{e}}}_{q})}_{x,y}{ {\mathcal F} }_{\parallel }(q),\,\,\hat{n}=(\begin{array}{cc}\sin \,\gamma  & {(-\mathrm{1)}}^{\chi }\,\cos \,\gamma \\ -\cos \,\gamma  & {(-\mathrm{1)}}^{\chi }\,\sin \,\gamma \end{array}),$$

where the real function ${ {\mathcal F} }_{\parallel }(q)$ does not depend on ***q*** direction ***e***_*q*_ = ***q***/*q*. Naturally, ${ {\mathcal F} }_{\parallel }(q)\propto q$ at *q* → 0.

When there is some spatial inhomogeneity of crystalline structure the electron gas acquires a local spin chirality. For an axially symmetric potential *V*(*r*) the excessive spin density *δ****S***(***r***) profile has a shape:

5 $$\delta {\boldsymbol{S}}({\boldsymbol{r}})=(\begin{array}{c}\delta {S}_{\parallel }(r)\cos (\chi {\phi }_{r}+\gamma ^{\prime} )\\ \delta {S}_{\parallel }(r)\sin (\chi {\phi }_{r}+\gamma ^{\prime} )\\ \delta {S}_{z}(r)\end{array}),\,\,\delta {S}_{z,\parallel }(r)=\int \,\frac{qdq}{2\pi }{J}_{\mathrm{0,1}}(qr){ {\mathcal F} }_{z,\parallel }(q)V(q),$$

where ***r*** = (*r*, *φ*_*r*_), $\delta {S}_{\parallel },\delta {S}_{z}$ depend on *r*, *J*_0,1_ are Bessel’s functions of the zeroth and first order, respectively, *γ*′ = *γ* + *π*/2. The emerging chiral spin cloud is similar to a skyrmion for *χ* = 1, or to an antiskyrmion for *χ* = −1. The fact that the helicity *γ*′ of the real space spin rotation is shifted by *π*/2 with respect to *γ* in *k*-space reflects the spin precession mechanism.

The details of chiral spin response naturally depend on a carrier band structure ${\varepsilon }_{k}^{\pm }$. To analyze general features of the studied phenomena we consider the two most widely used models with different particular symmetry properties. Namely, we calculate ${ {\mathcal F} }_{z,\parallel }(q)={ {\mathcal F} }_{z,\parallel }^{+}+{ {\mathcal F} }_{z,\parallel }^{-}$, which is a sum of the responses of two subbands (see Supplementary Appendix B), and analyze the electron spin density for parabolic-like and Dirac electron spectra.

## Parabolic-like Spectrum

**Figure 2:**
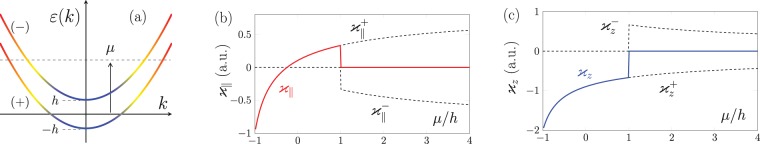
(**a**) Parabolic-like electron spectrum Eq. 6, (**b**,**c**) the dependence of ${\varkappa }_{\parallel ,z}$ on *μ* for *ξ* = 0.5.

Let us assume the following Hamiltonian *H*_*k*_ and the energy spectrum ${\varepsilon }_{k}^{s}$:

6 $${H}_{k}=\frac{{k}^{2}}{2m}-{{\boldsymbol{B}}}_{k}\cdot \hat{{\boldsymbol{\sigma }}},\,\,{\varepsilon }_{k}^{\pm }=\frac{{k}^{2}}{2m}\mp {B}_{k},\,\,{B}_{k}=\sqrt{{h}^{2}+{(\lambda k)}^{2}},$$

where *m* is the effective mass in the absence of *B*_*k*_ (we assume $\hslash$ = 1). In this paper we take the parameter *ξ* = *mλ*^2^/*h* < 1. The spectrum of the system is shown in Fig. 2a, the color within each subband indicates the magnitude of *ζ*_*s*_ = *λk*_*s*_/*h*, which has a meaning of spin inclination into the plane of the carrier motion (blue color corresponds to ${\zeta }_{s}\ll 1$, red color indicates ${\zeta }_{s}\gg 1$). We have obtained analytic expressions for the spin-density response functions ${ {\mathcal F} }_{z,\parallel }$ with the spectrum given by Eq. 6. As the formulas are rather cumbersome, we provide them in the Supplementary Appendix A (the details of the calculations are given in the Supplementary Appendix B). Importantly, ${ {\mathcal F} }_{z,\parallel }^{\pm }$ within each subband are decomposed into a sum of intra- and interband contributions ${ {\mathcal F} }_{z,\parallel }^{\pm }={ {\mathcal F} }_{z,\parallel }^{\pm \pm }+{ {\mathcal F} }_{z,\parallel }^{\pm \mp }$ with the interband terms exhibiting an additional coupling ${ {\mathcal F} }_{z}^{\pm \mp }(q)=(2m\lambda /q){ {\mathcal F} }_{\parallel }^{\pm \mp }(q)$.

Let us firstly consider the local coupling regime, when $\delta {S}_{x,y}({\boldsymbol{r}})={\varkappa }_{\parallel }{(\hat{n}\cdot \overrightarrow{\nabla })}_{x,y}V({\boldsymbol{r}})$ and $\delta {S}_{z}({\boldsymbol{r}})={\varkappa }_{z}V({\boldsymbol{r}})$. The coefficients ${\varkappa }_{z,\parallel }={\varkappa }_{z,\parallel }^{+}+{\varkappa }_{z,\parallel }^{-}$ found from the limiting behavior of ${ {\mathcal F} }_{z,\parallel }^{\pm }$ at *q* → 0 are given by

7 $${\varkappa }_{z}^{s}=-s\frac{m}{4\pi }\frac{{\rm{\Theta }}[\mu +sh]}{\sqrt{1+{\zeta }_{s}^{2}}-s\xi },\,\,{\varkappa }_{\parallel }^{s}=\frac{s}{8\pi \lambda }(1-\frac{1}{\sqrt{1+{\zeta }_{s}^{2}}-s\xi }){\rm{\Theta }}[\mu +sh\mathrm{]}.$$

Let us mention that the coefficient ${\varkappa }_{z}^{\pm }$ is the product of the electron density of states and *z*-projection of spin taken at the Fermi energy *μ*. The dependence of ${\varkappa }_{z,\parallel }$ on *μ* is shown in Fig. 2b,c. We note that nonzero spin response is observed only when the upper subband is empty (*μ* < *h*). This result is an inherent property of the considered model; the background spin density ${S}_{z}^{0}=(mh/4\pi )$ remains constant at *μ* > *h*.

As follows from the explicit expressions for ${ {\mathcal F} }_{z,\parallel }^{\pm }$, the local coupling regime occurs when the Fourier components of *V*(***q***) are localized within $q\ll \,{\rm{\min }}\,[{k}_{\pm },{a}_{0}^{-1}]$, where *a*_0_ = *λ*/2*h*. For these values the response functions ${ {\mathcal F} }_{z}$, ${ {\mathcal F} }_{\parallel }/q$ have a weak dependence on *q*, which means no spatial dispersion and, thus, the absence of non-locality in the response. Note, that, apart from the Fermi wavevector *k*_±_, there is a second spatial scale *a*_0_ = *λ*/2*h*, which controls the spatial dispersion of the spin response. This scale is associated with the precession mechanism for the in-plane spin generation.

**Figure 3:**
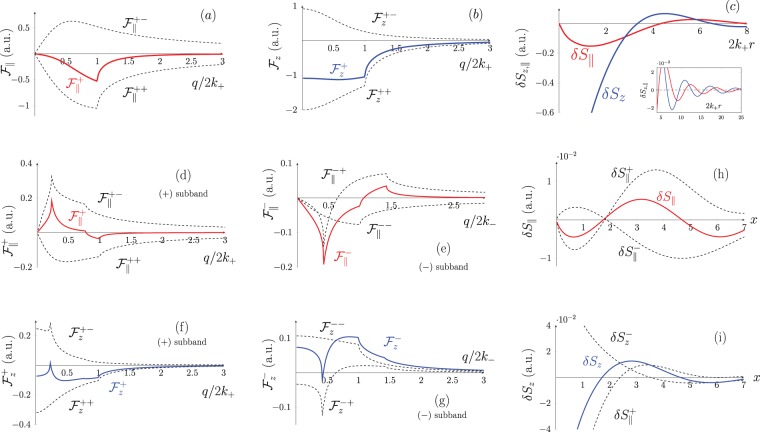
(**a**–**c**) The dependence of ${ {\mathcal F} }_{z,\parallel }^{+}$ on *q*/2*k*_+_, and $\delta {S}_{z,\parallel }$ on 2*k*_+_*r* in case of one filled spin subband (*ξ* = 0.5, *μ* = −0.4*h*, *ζ*_+_ = 1), (**d**–**g**) the dependence of ${ {\mathcal F} }_{z,\parallel }^{\pm }$ on *q*/2*k*_±_, (**h**,**i**) the dependence of $\delta {S}_{z,\parallel }$ on $x\mathrm{=2(}\sqrt{2m\mu })r$ in case of two filled spin subbands, the parameters are *μ* = 3.5*h*, *ξ* = 0.5, *ζ*_+_ = 2.5, *ζ*_−_ = 1.4.

We proceed with considering the non-local spin response, firstly for the case when only the (+) subband is populated (−*h* < *μ* < *h*). The dependence of ${ {\mathcal F} }_{z,\parallel }^{+}$, and its partial contributions ${ {\mathcal F} }_{z,\parallel }^{+\pm }$ on *q*/2*k*_+_ is shown in Fig. 3(a,b). As we have discussed above, the response functions at *q* → 0 behave as ${ {\mathcal F} }_{z}^{+}\approx {\varkappa }_{z}^{+}$, ${ {\mathcal F} }_{\parallel }^{+}\approx {\varkappa }_{\parallel }^{+}q$. Another general trend is that the intra-${ {\mathcal F} }_{z,\parallel }^{++}$ and interband ${ {\mathcal F} }_{z,\parallel }^{+-}$ terms have an opposite sign and, thus, tend to cancel each other. The spatial profile $\delta {S}_{z,\parallel }(r)$ induced around a repulsive short range potential *V*(*r*) = *α*_0_*δ*(***r***) is shown in Fig. 3c. The largest spin response appears within the Fermi wavelength ($2{k}_{+}r\lesssim 2$). Far apart from the center ${\delta }{S}_{z,\parallel }(r)$ decrease exhibiting the Friedel oscillations with the period 2*k*_+_ (see inset in Fig. 3).

Let us now consider the case *μ* > *h* when both spin subbands are populated. Although the local spin response is absent in this case (${\varkappa }_{z,\parallel }=\mathrm{0)}$, the effect of spatial dispersion restores the chiral spin pattern. Shown in Fig. 3(d–g) are the obtained spin response functions ${ {\mathcal F} }_{z,\parallel }^{\pm }(q)$. We note that the intraband terms ${ {\mathcal F} }_{z,\parallel }^{\pm \pm }$ for both subbands exhibit a single spike at *q* = 2*k*_±_. What is more interesting is the double-spike structure of the interband terms ${ {\mathcal F} }_{z,\parallel }^{\pm \mp }$ driven by two nesting vectors connecting two distinct subbands of the Fermi surface. The presence of the two different spatial scales *k*_±_ along with a complex structure of interband transitions lead to quite a peculiar spin response in the real space. In Fig. 3h,i we demonstrate $\delta {S}_{z,\parallel }(r)$ for the short range potential *V*(*q*) = *α*_0_. The spatial scale is given in units of *x* = *k*_*F*_*r*, where *k*_*F*_ is the averaged Fermi wavevector. The Friedel oscillations clearly visible at large distances from the centre $x\gg 1$ are now formed by the superposition of oscillations with different spatial periods.

## Dirac Spectrum

Let us further consider the case of Hamiltonian with Dirac spectrum:

8 $${H}_{k}=-\,{{\boldsymbol{B}}}_{k}\cdot \hat{{\boldsymbol{\sigma }}},\,{\varepsilon }_{k}^{\pm }=\mp \,\sqrt{{h}^{2}+{(\lambda k)}^{2}}.$$

**Figure 4:**
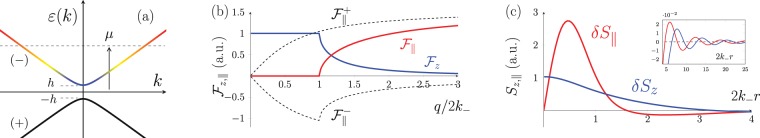
(**a**) Dirac electron spectrum, (**b**) the dependence of ${ {\mathcal F} }_{z,\parallel }^{\pm }$ on *q*/2*k*_−_, (**c**) the dependence of $\delta {S}_{\parallel ,z}$ on 2*k*_−_*r*, the inset shows the Friedel’s oscillations. The parameters are *μ* = 2*h*, *ζ*_−_ = 1.7, *ak*_−_ = 0.3.

This model describes, for example, chiral surface states of a 3D TI (the SOI parameters are *χ* = 1, *γ* = *π*). Shown in Fig. 4a is the spectrum (8), which consists of two nearly linear bands separated by the gap 2*h*. The fundamental difference from the previously considered parabolic-like spectrum is the additional electron-hole symmetry (${\mathscr{C}}$-symmetry), which modifies the electron gas response to external perturbations^42–45^.

Let us put the Fermi energy *μ* > 0 above the charge neutrality point, so the lower (+) subband is completely filled while the upper (−) subband is filled partially. Our calculations show that the (+) subband does not contribute to the response of *z* spin component (${ {\mathcal F} }_{z}^{+}=0$), while for the (−) subband the spin response function is the conventional 2D Lindhard function (the details of the calculations are given in the Supplementary Appendix B):

9 $${ {\mathcal F} }_{z}^{-}(q)=\frac{{m}_{g}}{4\pi }(1-{\rm{\Theta }}[q-2{k}_{-}]\sqrt{1-4{k}_{-}^{2}/{q}^{2}}),$$

where *m*_*g*_ = *h*/*λ*^2^ is an effective mass due to the spectrum gap, ${k}_{-}=\sqrt{{\mu }^{2}-{h}^{2}}/\lambda$ is the Fermi wavevector. This result is rather interesting as the Lindhard function usually describes the susceptibility of a system with simple parabolic spectrum.

Another important feature of ${ {\mathcal F} }_{z}^{-}$ given by (9) is that its magnitude does not depend on the Fermi energy *μ*. This is in contrast with the parabolic-like case, where the increase of *μ* leads to the suppression of spin response according to ${ {\mathcal F} }_{z}^{\pm }\propto \mathrm{1/}{\zeta }_{\pm }$ at ${\zeta }_{\pm }\gg 1$. This effect is due to the density of states, which for the Dirac spectrum takes the form *ν*_−_(*µ*) = *µ*/2*πλ*^2^. Upon the increase of the Fermi energy the suppression of spin *z*-projection (which is *h*/2*B*_*k*_ ∝ 1/*ζ*_−_ at $\mu \gg h$) is exactly compensated by the increase of *ν*_−_(*μ*). For instance, considering the local coupling regime $\delta {S}_{z}={\varkappa }_{z}V({\boldsymbol{r}})$ the spin response is exactly determined by a product ${\varkappa }_{z}^{-}={\nu }_{-}(\mu ){h}/2\mu ={m}_{g}\mathrm{/4}\pi$ independent of *μ*.

The in-plane spin response also exhibits a number of peculiar features. For the functions ${ {\mathcal F} }_{\parallel }^{\pm }(q)$ we obtained:

10 $${ {\mathcal F} }_{\parallel }^{+}(q)=\frac{{m}_{g}}{4\pi }{\tan }^{-1}(q{a}_{0}),\,\,{ {\mathcal F} }_{\parallel }^{-}(q)=-\,{ {\mathcal F} }_{\parallel }^{+}(q)+\frac{{m}_{g}}{4\pi }{\rm{\Theta }}[q-2{k}_{-}]{\tan }^{-1}({a}_{0}\sqrt{\frac{{q}^{2}-4{k}_{-}^{2}}{1+{\zeta }_{-}^{2}}}).$$

We note that there is a non-zero spin response from the completely filled (+) subband, and that ${ {\mathcal F} }_{\parallel }^{+}/q$ remains finite even at $q{a}_{0}\ll 1$. This is an unusual behavior, since no charge density response of the (+) subband can be induced in this case. Indeed, the interband transitions underlying the change of electron density are suppressed for a smooth potential $q{a}_{0}\ll 1$ due to the finite band gap 2*h*. On the contrary, the in-plane spin response originates from the spin precession driven by a drift electron flow, which remains finite in ${\mathscr{C}}$-symmetry systems even with gaped spectrum due to the Klein tunneling.

Considering the in-plane spin response from the upper (−) subband we note that the function ${ {\mathcal F} }_{\parallel }^{-}(q)$ given by Eq. 10 contains both the *μ*-independent term opposite to that of (+) subband, and a *μ*-aware contribution responsible for the Friedel’s oscillations with the spatial period 2*k*_−_. The in-plane spin response function ${ {\mathcal F} }_{\parallel }={ {\mathcal F} }_{\parallel }^{+}+{ {\mathcal F} }_{\parallel }^{-}$ and its partial components ${ {\mathcal F} }_{\parallel }^{\pm }$ are shown in Fig. 4b. The contributions of (±) subbands cancel each other at *q* < 2*k*_−_ and ${ {\mathcal F} }_{\parallel }$ turns to zero. Therefore, no in-plane spin response is induced by a long-range electrostatic perturbation when the Fermi level is in the upper subband.

The non-local spin response is also modified due to ${\mathscr{C}}$-symmetry. As can be seen in Fig. 4b the function ${ {\mathcal F} }_{\parallel }(q)={ {\mathcal F} }_{\parallel }^{+}+{ {\mathcal F} }_{\parallel }^{-}$ saturates at $q\gg {k}_{-}$ instead of going to zero. However, as discussed above, the in-plane spin density responds to the potential gradient, so it is ${ {\mathcal F} }_{\parallel }(q)/q$ which has the physical meaning and it indeed decays as 1/*q* at *q* → ∞. In Fig. 4c we show the spatial spin pattern $\delta {S}_{z,\parallel }$ induced by a short-range potential *V*(*q*) = *α*_0_e*xp*[−(*qa*/2)^2^], *a* is the potential radius. It is worth mentioning that the magnitude of the in-plane spin response $\delta {S}_{\parallel }$ in the vicinity of a defect is far larger than in the parabolic spectra case due to the saturation of ${ {\mathcal F} }_{\parallel }(q)$. This finding emphasizes a particularly high susceptibility of a chiral spin pattern in response to an electrostatic disorder in systems with Dirac spectrum.

## Summary and Discussion

Our study suggests that the emergence of chiral spin textures in an electron gas driven by an electrostatic disorder is a universal phenomenon, which is expected in a variety of experimentally studied systems, such as DMS^40,46^, thin films of ferromagnets^32,33^, Bi_2_Se_3_ doped by magnetic impurities^30,31,47,48^, or due to the proximity effect^49^ with magnetic insulators^50^, or ferromagnets^51,52^. The derived analytical expressions for the spin-density response functions can be used to qualitatively analyze the effect in these systems and to treat the interplay between the spin-orbit interaction and Zeeman spin splitting of carriers in particular. The latter is of special importance for the locality of spin response, as the theory reveals the existence of an additional universal scale *a*_0_ = *λ*/2*h*, which triggers the spatial dispersion.

We believe, that there are diverse experimental approaches allowing to reveal the chiral perturbation of the 2DEG spin density. For instance, probing the chiral spin textures induced on a surface by means of spin-polarized scanning tunneling microscopy^53^ would be a new tool to access the parameters of the electron gas. The chiral spin pattern in the 2DEG can also induce a chiral order of magnetic ions due to the exchange interaction between these subsystems. In particular, this effect can occur not only for the magnetic moments located in the same material, but also for those in a different layer of a heterostructure due to the proximity effect^49^. Thus a chiral ordering of the magnetization can be induced in various systems by electrical means via the modulation of 2DEG spin density, hence, being an alternative tool to create magnetic skyrmions and other chiral spin textures. Also, the considered mechanism is likely to be responsible for the recently observed topological Hall effect in TI and DMS^54–56^. Indeed, the topological Hall effect originates from the asymmetric scattering of electrons on chiral spin textures^16^. It has been shown that it is not limited to magnetic skyrmions possessing a topological charge but also expected for a broad range of chiral spin textures^16,57^. One possible scenario for the formation of such chiral spin textures in magnetic systems with spin-orbit interaction is suggested by the presented theory. As follows from our study, the chiral spin textures inevitably emerge nearby defects and other inhomogeneities of a real structure and eventually they can give rise to the topological Hall effect.

## Supplementary information

Spin-density correlation functions
