# Supplementary material for: Chiral spin ordering of electron gas in solids with broken time reversal symmetry
Source: Sci Rep. 2019 Jul 25;9:10817. doi: 10.1038/s41598-019-47274-6 (PMC6658505; doi:10.1038/s41598-019-47274-6)
Supplement: Supplementary file 1 — Spin-density correlation functions [file 41598_2019_47274_MOESM1_ESM.pdf]

Chiral spin ordering of electron gas in solids with broken time reversal symmetry - Supplemental Materials

K. S. Denisov,[1,2] I. V. Rozhansky,[1,2] N. S. Averkiev,[1] and E. Lähderanta[2]

[1] *Ioffe Institute, 194021 St.Petersburg, Russia*

[2] *Lappeenranta-Lahti University of Technology, FI-53851 Lappeenranta, Finland*

Appendix A: The spin-density response functions for the parabolic-like spectrum.

Here we provide the derived analytical formulas for the spin-density response functions $\mathcal{F}^{\pm}_{z,\parallel}(q)$ in case of the electron parabolic-like spectrum $\varepsilon^{\pm}_k = k^2/2m \mp \sqrt{h^2+(\lambda k)^2}$ (see Fig. 1a, Eq. 6 and the notation used in the main text). In the formulas below we use the following parameters: $\xi = m\lambda^2/h < 1$, $\zeta_\pm = \lambda k_\pm/h$, $k_\pm$ is the Fermi wavevector in the corresponding subband, $a_0 = \lambda/2h$ has a dimensionality of length. We find that $\mathcal{F}^{\pm}_{z,\parallel}(q)$ are decomposed onto the intra- and interband contributions as $\mathcal{F}^{\pm}_{z,\parallel} = \mathcal{F}^{\pm\pm}_{z,\parallel} + \mathcal{F}^{\pm\mp}_{z,\parallel}$. The intraband terms $\mathcal{F}^{\pm\pm}_{z,\parallel}(q)$ are given by:

$$\begin{aligned}
&\mathcal{F}^{\pm\pm}_{\parallel}(q) = \frac{m}{4\pi}\frac{1}{y(q)}\Big(\Theta[2k_\pm - q]\Phi_1(q) + \Theta[q-2k_\pm]\Phi^{\pm}_2(q) - \Phi^{\pm}_3(q)\Big),\\
&y(q) = \sqrt{1+(qa_0)^2-\xi^2}, \qquad \Phi_1(q) = \ln\sqrt{1+(qa_0)^2}, \qquad \Phi^{\pm}_3(q) = \tanh^{-1}\left(\frac{qa_0}{y(q)}\right) + \ln\frac{y(q)\pm\xi(qa_0)}{\sqrt{1-\xi^2}},\\
&\Phi^{\pm}_2(q) = \tanh^{-1}\left(\frac{a_0}{y(q)}\sqrt{q^2-4k^2_\pm}\right) + \ln\left[y(q)\sqrt{1+\zeta^2_\pm} \pm \xi a_0\sqrt{q^2-4k^2_\pm}\right] - \frac{1}{2}\ln\left[1+\zeta^2_\pm-\xi^2\right].
\end{aligned} \tag{A1}$$

$$\begin{aligned}
&\mathcal{F}^{\pm\pm}_{z}(q) = \mp\frac{m}{4\pi}\frac{1}{y(q)}\frac{\xi}{qa_0}\Big(\Theta[2k_\pm - q]\Lambda^{\pm}_1(q) + \Theta[q-2k_\pm]\Lambda^{\pm}_2(q) - \Lambda^{\pm}_3(q)\Big),\\
&\Lambda^{\pm}_1(q) = y(q)\frac{\pi}{2\xi} \mp \ln\sqrt{1+(qa_0)^2}, \quad \Lambda^{\pm}_3(q) = \frac{y(q)}{\xi}\tan^{-1}\left(\frac{1}{qa_0}\right) \mp \ln\left[1+qa_0\frac{y(q)+qa_0}{1\mp\xi}\right],\\
&\Lambda^{\pm}_2(q) = \frac{y(q)}{\xi}\tan^{-1}\left(\frac{1}{a_0}\sqrt{\frac{1+\zeta^2_\pm}{q^2-4k^2_\pm}}\right) \mp \left(\ln\left[1+(qa_0)^2 + y(q)a_0\sqrt{q^2-4k^2_\pm} \mp \xi\sqrt{1+\zeta^2_\pm}\right] - \ln\left[\sqrt{1+\zeta^2_\pm}\mp\xi\right]\right),
\end{aligned} \tag{A2}$$

and $\Theta[x]$ is the Heaviside function. The interband terms experience an additional symmetry $\mathcal{F}^{\pm\mp}_z = \mathcal{F}^{\pm\mp}_{\parallel}(2m\lambda/q)$. The functions $\mathcal{F}^{\pm\mp}_{\parallel}(q)$ are given by:

$$\begin{aligned}
&\mathcal{F}^{+-}_{\parallel}(q) = \frac{m}{4\pi}\frac{1}{y(q)} \times \begin{cases} \mathcal{J}_+(q,1), & \zeta_+ < 2\sqrt{\xi+\xi^2} \\ \Theta[q^+_1 - q]\mathcal{J}_+(q,1) + \Theta[q-q^+_1]\Theta[q^+_2-q]\mathcal{J}_+(q,x_+(q)) + \Theta[q-q^+_2]\mathcal{J}_+(q,1), & \zeta_+ > 2\sqrt{\xi+\xi^2}\end{cases}\\
&\mathcal{F}^{-+}_{\parallel}(q) = \frac{m}{4\pi}\frac{1}{y(q)}\Big(\Theta[q^-_1 - q]\mathcal{J}_-(q,1) + \Theta[q-q^-_1]\Theta[q^-_2-q]\mathcal{J}_-(q,|x_-(q)|) + \Theta[q-q^-_2]\mathcal{J}_-(q,1)\Big),\\
&\mathcal{J}_\pm(q,x) = [\mathrm{sgn}(q-q_0)]^{\frac{(1\mp1)}{2}}\ln\left[\frac{1+\sqrt{F_\pm(q,0)}}{1+\sqrt{F_\pm(q,x)}}\frac{\Delta_\pm(x)}{\Delta_\pm(0)}\right], \qquad q_0 = \frac{1}{a_0}\sqrt{\xi+\xi^2},\\
&F_\pm(q,x) = 1+\xi\left(\frac{\Delta_\pm(x)}{y(q)}\right)^2\left[\left(\frac{2k_\pm}{q}\right)^2 - \left(\frac{\zeta_\pm}{\xi}\right)^2\right], \qquad \Delta_\pm(x) = \frac{\xi}{\zeta^2_\pm}\left[\xi\mp\sqrt{1+\zeta^2_\pm x^2}\right],\\
&q^{\pm}_{1,2} = k_\pm\left[(-1)^{1,2}\right]^{\frac{(1\mp1)}{2}}\left(1+(-1)^{1,2}\sqrt{1+4\Delta_\pm(1)}\right), \qquad x_\pm(q) = \mathrm{Re}\left[\frac{q}{2k_\pm} \pm \frac{2m\lambda}{\zeta_\pm}\frac{y(q)}{\sqrt{q^2-(2m\lambda)^2}}\right].
\end{aligned} \tag{A3}$$

The dependence of $\mathcal{F}^{\pm}_{z,\parallel}(q)$ on q is shown in Fig. 2 in these Materials and is thoughtfully discussed in the main text. In the limit of $q \to 0$ these functions behave as $\mathcal{F}^{\pm}_z(q) \approx \varkappa^{\pm}_z$, and $\mathcal{F}^{\pm}_{\parallel}(q) \approx q\cdot\varkappa^{\pm}_{\parallel}$, the coefficients $\varkappa^{\pm}_{z,\parallel}$ describing the local coupling regime are found to be:

$$\varkappa^{\pm}_z = \mp\frac{m}{4\pi}\frac{\Theta[\mu\pm h]}{\sqrt{1+\zeta^2_\pm}\mp\xi}, \qquad \varkappa^{\pm}_{\parallel} = \pm\frac{1}{8\pi\lambda}\left(1-\frac{1}{\sqrt{1+\zeta^2_\pm}\mp\xi}\right)\Theta[\mu\pm h]. \tag{A4}$$

Appendix B: Derivation of spin-density response functions.

We consider a two-dimensional electron gas with an effective magnetic field acting on an electron spin in k-space: $\boldsymbol{B}_k = (\lambda k \cos(\chi\varphi_k + \gamma), \lambda k \sin(\chi\varphi_k + \gamma), h)$, where $(\lambda, h) > 0$, $\chi = \pm 1$, γ is an arbitrary real number. There are two spin subbands $s = \pm$, an electron in state $(\boldsymbol{k}, s)$ has its spin $\boldsymbol{S}_k^{\pm}$ parallel ($s = +$) or antiparallel ($s = -$) to $\boldsymbol{B}_k$. The corresponding spinors $|u_k^s\rangle$ for $(\boldsymbol{k}, s)$ states are given:

$$|u_k^+\rangle = \begin{pmatrix} e^{-i(\chi\varphi_k+\gamma)} a_k \\ b_k \end{pmatrix}, \qquad |u_k^-\rangle = \begin{pmatrix} b_k \\ -e^{i(\chi\varphi_k+\gamma)} a_k \end{pmatrix}, \tag{B1}$$
$$a_k = \sqrt{\frac{1+n_k}{2}} = \frac{h + B_k}{\sqrt{(\lambda k)^2 + (h+B_k)^2}}, \qquad b_k = \sqrt{\frac{1-n_k}{2}} = \frac{\lambda k}{\sqrt{(\lambda k)^2 + (h+B_k)^2}},$$

where $n_k = h/B_k$, and $B_k = \sqrt{h^2 + (\lambda k)^2}$. The static spin-density response functions $\mathcal{F}_\alpha(\boldsymbol{q})$ introduced in the main text Eq. 2 contain contributions from each spin subband and are given by:

$$\mathcal{F}_\alpha(\boldsymbol{q}) = \mathcal{F}_\alpha^+(\boldsymbol{q}) + \mathcal{F}_\alpha^-(\boldsymbol{q}), \qquad \mathcal{F}_\alpha^s(\boldsymbol{q}) = \sum_{k,s'=\pm} f_k^s \left(\frac{\langle u_k^s|\hat{S}_\alpha|u_{k+q}^{s'}\rangle\langle u_{k+q}^{s'}|u_k^s\rangle}{\varepsilon_k^s - \varepsilon_{k+q}^{s'} + i0} + \frac{\langle u_{k-q}^{s'}|\hat{S}_\alpha|u_k^s\rangle\langle u_k^s|u_{k-q}^{s'}\rangle}{\varepsilon_k^s - \varepsilon_{k-q}^{s'} - i0} \right), \tag{B2}$$

where $\hat{S}_\alpha = \hat{\sigma}_\alpha/2$, $\hat{\sigma}_\alpha$ is the Pauli matrix, $\alpha = (x, y, z)$, f_k^s and ε_k^s are the distribution function and an electron energy in state $(\boldsymbol{k}, s)$. Replacing $\boldsymbol{k} \to -\boldsymbol{k}$ in the last integral (we assume that $\varepsilon_k^s = \varepsilon_{-k}^s$) and using the following relations:

$$\langle u_{-k-q}^{s'}|\hat{S}_z|u_{-k}^s\rangle\langle u_{-k}^s|u_{-k-q}^{s'}\rangle = \langle u_{k+q}^{s'}|\hat{S}_z|u_k^s\rangle\langle u_k^s|u_{k+q}^{s'}\rangle, \tag{B3}$$
$$\langle u_{-k-q}^{s'}|\hat{S}_{x,y}|u_{-k}^s\rangle\langle u_{-k}^s|u_{-k-q}^{s'}\rangle = -\langle u_{k+q}^{s'}|\hat{S}_{x,y}|u_k^s\rangle\langle u_k^s|u_{k+q}^{s'}\rangle,$$

we get for the response functions at zero temperature:

$$\mathcal{F}_\alpha^s(\boldsymbol{q}) = \int_0^{k_s} \frac{kdk}{2\pi} \mathcal{P} \int_0^{2\pi} \frac{d\theta}{2\pi} \sum_{s'=\pm} \frac{\mathcal{L}_{\alpha,kq}^{ss'}}{\varepsilon_k^s - \varepsilon_{k+q}^{s'}}, \tag{B4}$$
$$\mathcal{L}_{z,kq}^{ss'} = \mathrm{Re}\left[\langle u_k^s|\hat{\sigma}_z|u_{k+q}^{s'}\rangle\langle u_{k+q}^{s'}|u_k^s\rangle\right], \quad \mathcal{L}_{xy,kq}^{ss'} = i \times \mathrm{Im}\left[\langle u_k^s|\hat{\sigma}_{x,y}|u_{k+q}^{s'}\rangle\langle u_{k+q}^{s'}|u_k^s\rangle\right],$$

where $\mathcal{P}$ stands for the principal value, θ is the polar angle between $\boldsymbol{k}$ and $\boldsymbol{q}$, $k_\pm$ is the Fermi wavevector in s subband. We note that $\mathcal{L}_{xy,kq}^{ss'}$ are purely imaginary. It follows from the structure of $\mathcal{L}_{xy,kq}^{ss'}$, that the response functions $\mathcal{F}_{x,y}^s(\boldsymbol{q})$ for the in-plane spin components can be presented in form:

$$\mathcal{L}_{xy,kq}^{ss'} = i\left(\hat{n}\boldsymbol{e}_q\right)_{x,y} \mathcal{L}_{\parallel,kq}^{ss'}, \qquad \mathcal{F}_{x,y}^s(\boldsymbol{q}) = i(\hat{n}\boldsymbol{e}_q)_{x,y}\mathcal{F}_\parallel^s(q), \tag{B5}$$
$$\mathcal{F}_\parallel^s(q) = \int_0^{k_s} \frac{kdk}{2\pi} \mathcal{P} \int_0^{2\pi} \frac{d\theta}{2\pi} \sum_{s'=\pm} \frac{\mathcal{L}_{\parallel,kq}^{ss'}}{\varepsilon_k^s - \varepsilon_{k+q}^{s'}}, \qquad \hat{n} = \begin{pmatrix} \sin\gamma & (-1)^\chi \cos\gamma \\ -\cos\gamma & (-1)^\chi \sin\gamma \end{pmatrix}, \tag{B6}$$

where $\hat{n}$ is an orthogonal matrix determined by a particular spin-orbit interaction type, $\boldsymbol{e}_q = \boldsymbol{q}/q$ is the unit vector in the direction of $\boldsymbol{q}$, the functions $\mathcal{F}_\parallel^\pm(q)$ are real and depend only on q modulus. The explicit expressions for the matrix elements $\mathcal{L}_{z,\parallel,kq}^{ss'}$ are given:

$$\mathcal{L}_{z,kq}^{ss'}, = \frac{(-1)^{\frac{(1-s)}{2}}}{2}(n_k + (-1)^{s-s'} n_{k+q}), \qquad \mathcal{L}_{\parallel,kq}^{ss'}, = (-1)^{s-s'}(qa_0)n_k n_{k+q}, \qquad a_0 = \frac{\lambda}{2h}. \tag{B7}$$

When calculating these matrix elements the following relation is very useful: $(a_k b_k)/(a_k^2 - b_k^2) = k a_0$. The details of the spin-density response depend on a particular electron spectrum, below we provide the calculations for parabolic-like and Dirac types of spectra.

1. Integrals for the Dirac spectrum

Here we present the calculations of $\mathcal{F}^{\pm}_{z,\|}(q)$ for the Dirac electron spectrum $\varepsilon^{\pm}_k = \mp B_k = \mp\sqrt{h^2+(\lambda k)^2}$, see Eq. 7 in the main text and Fig.3a. We write the spin-density response functions in form:

$$\begin{pmatrix}\mathcal{F}^s_{\|}(q)\\ \mathcal{F}^s_z(q)\end{pmatrix} = -\int\limits_0^{k_s}\frac{kdk}{2\pi}\mathcal{P}\int\limits_0^{2\pi}\frac{d\theta}{2\pi}\begin{pmatrix}s\mathcal{A}_{k,q}\times(qa_0)\\ \mathcal{B}_{k,q}\end{pmatrix}, \tag{B8}$$

$$\mathcal{A}_{k,q} = \left[\frac{1}{B_k - B_{k+q}} - \frac{1}{B_k+B_{k+q}}\right]n_k n_{k+q} = -\frac{1}{q}\left(\frac{m_g}{B_k}\right)\frac{2h}{q+2k\cos\theta},$$

$$\mathcal{B}_{k,q} = \frac{1}{2}\left[\frac{n_k+n_{k+q}}{B_k-B_{k+q}} + \frac{n_k-n_{k+q}}{B_k+B_{k+q}}\right] = -\frac{2h}{q+2k\cos\theta},$$

where we introduced the parameter $m_g = h/\lambda^2$, which is an effective mass at the bottom of subband $k\approx 0$. The integral over the angle in Eq. B8 for both $\mathcal{F}^s_{z,\|}$ is taken using:

$$\mathcal{P}\int\limits_0^{2\pi}\frac{d\theta}{2\pi}\frac{1}{a+b\cos\theta} = \frac{\Theta[a-b]}{\sqrt{a^2-b^2}}, \qquad a,b>0. \tag{B9}$$

The remaining integrals over k are taken using I_z for $\mathcal{F}^s_z$, and $I_\|$ for $\mathcal{F}^s_\|$ correspondingly:

$$I_z = \int\limits_0^1 xdx\frac{\Theta[z-x]}{\sqrt{z^2-x^2}} = z - \Theta[z-1]\sqrt{z^2-1}, \tag{B10}$$

$$I_\| = \int\limits_0^1 xdx\frac{1}{\sqrt{y^2+x^2}}\frac{\Theta[z-x]}{\sqrt{z^2-x^2}} = \tan^{-1}\left(\frac{z}{y}\right) - \Theta[z-1]\tan^{-1}\left(\sqrt{\frac{z^2-1}{y^2+1}}\right).$$

Let us consider the case when the Fermi energy $\mu > h$ lies in the upper subband (see Fig.3a). The response from partially filled ($-$) subband, using the integrals in Eq. (B9,B10), is given by:

$$\mathcal{F}^-_z(q) = \frac{m_g}{4\pi}\left(1-\Theta[q-2k_-]\sqrt{1-(q/2k_-)^2}\right),$$

$$\mathcal{F}^-_\|(q) = -\frac{m_g}{4\pi}\left[\tan^{-1}(qa_0) - \Theta[q-2k_-]\tan^{-1}\left(a_0\sqrt{\frac{q^2-4k_-^2}{1+\zeta_-^2}}\right)\right], \tag{B11}$$

here $\zeta_- = \lambda k_-/h$. Considering the response from the fully filled (+) subband we should take the integrals Eq. B10 in the limit $k_+\to\infty$. At that no response of spin z-component is induced ($\mathcal{F}^+_z = 0$), while the in-plane response function is given by:

$$\mathcal{F}^+_\|(q) = \frac{m_g}{4\pi}\tan^{-1}(qa_0). \tag{B12}$$

2. Integrals for the parabolic-like spectrum

Here we present the calculations of $\mathcal{F}^{\pm}_{z,\parallel}(q)$ for the parabolic-like electron spectrum $\varepsilon^{\pm}_k = k^2/2m \mp \sqrt{h^2+(\lambda k)^2}$, see Eq. 6 in the main text and Fig.1a. We write the spin-density response functions in form:

$$\begin{pmatrix}\mathcal{F}^s_\parallel(q)\\ \mathcal{F}^s_z(q)\end{pmatrix} = \int\limits_0^{k_s}\frac{kdk}{2\pi}\mathcal{P}\int\limits_0^{2\pi}\frac{d\theta}{2\pi}\begin{pmatrix}\mathcal{C}^s_{k,q}\times(qa_0)\\ \mathcal{D}^s_{k,q}\end{pmatrix}, \tag{B13}$$

$$\mathcal{C}^{\pm}_{k,q} = \left[\frac{1}{(\delta\varepsilon_{k,q}\mp B_k)\pm B_{k+q}} - \frac{1}{(\delta\varepsilon_{k,q}\mp B_k)\mp B_{k+q}}\right]n_k n_{k+q} = \mp\frac{2hn_k}{(\delta\varepsilon_{k,q}\mp B_k)^2 - B^2_{k+q}}$$

$$\mathcal{D}^{\pm}_{k,q} = \pm\frac{1}{2}\left[\frac{n_k+n_{k+q}}{(\delta\varepsilon_{k,q}\mp B_k)\pm B_{k+q}} + \frac{n_k-n_{k+q}}{(\delta\varepsilon_{k,q}\mp B_k)\mp B_{k+q}}\right] = \frac{\pm n_k\delta\varepsilon_{k,q} - 2h}{(\delta\varepsilon_{k,q}\mp B_k)^2 - B^2_{k+q}},$$

$$\delta\varepsilon_{k,q} = -\frac{q^2}{2m} - \frac{kq}{m}\cos\theta.$$

The denominator in the $\mathcal{C}^{\pm}_{k,q}, \mathcal{D}^{\pm}_{k,q}$ can be expressed as $(\delta\varepsilon_{k,q}\mp B_k)^2 - B^2_{k+q} = (kq/m)^2\times(a_\pm + b_\pm\cos\theta + \cos^2\theta)$, where the coefficients $a_\pm, b_\pm$ do not depend on θ. There are two types of integrals with respect to the angle θ:

$$I^{\pm}_1 = \mathcal{P}\int\limits_0^{2\pi}\frac{d\theta}{2\pi}\frac{1}{a_\pm + b_\pm\cos\theta + \cos^2\theta} = \pm\frac{x^2 t}{|\Delta_\pm(x)|}\left[\frac{1}{w(t,x)} - \frac{\mathrm{sgn}\,(u_\pm(x,t))}{w(u_\pm,x)}\right], \tag{B14}$$

$$I^{\pm}_2 = \mathcal{P}\int\limits_0^{2\pi}\frac{d\theta}{2\pi}\frac{\cos\theta}{a_\pm + b_\pm\cos\theta + \cos^2\theta} = \pm\frac{xt}{|\Delta_\pm(x)|}\left[\mathrm{sgn}(u_\pm(x,t))\frac{u_\pm(x,t)}{w(u_\pm,x)} - \frac{t}{w(t,x)}\right], \tag{B15}$$

where we introduced the following notation: $x = k/k_\pm$, $t = q/2k_\pm$, the functions $\Delta_\pm(x), u_\pm(x,t), w(t,x)$ are given by:

$$\Delta_\pm(x) = \frac{\xi}{\zeta^2_\pm}\left[\xi \mp \sqrt{1+x^2\zeta^2_\pm}\right] \lessgtr 0, \quad u_\pm(x,t) = t - \frac{\Delta_\pm(x)}{t}, \quad w(t,x) = \mathrm{Re}\left[\sqrt{t^2-x^2}\right] = \Theta\left(|t|-x\right)\sqrt{t^2-x^2},$$

here $\xi = m\lambda^2/h$, $\zeta_\pm = \lambda k_\pm/h$. For the calculation of $\mathcal{F}^{\pm}_\parallel$ we need only $I^{\pm}_1$ integrals, while calculating $\mathcal{F}^{\pm}_z$ requires both $I^{\pm}_{1,2}$. After the integration over the angle we can decompose the integrals over k as $\mathcal{F}^{\pm}_{\parallel,z}(t) = \mathcal{F}^{\pm\pm}_{\parallel,z}(t) + \mathcal{F}^{\pm\mp}_{\parallel,z}(t)$, where

$$\mathcal{F}^{\pm\pm}_\parallel(t) = -Q_\pm\mathcal{I}^{\pm}_1, \qquad \mathcal{F}^{\pm\pm}_z(t) = \mp Q_\pm\frac{1}{qa_0}\mathcal{I}^{\pm}_2, \qquad \mathcal{F}^{\pm\mp}_\parallel(t) = \left(\frac{q}{2m\lambda}\right)\mathcal{F}^{\pm\mp}_z(t) = Q_\pm\mathcal{I}^{\pm}_3, \tag{B16}$$

here the pre-factor $Q_\pm = (m/4\pi)(m\lambda/k_\pm)$, the integrals $\mathcal{I}^{\pm}_{1,2,3}$ are shown below:

$$\mathcal{I}^{\pm}_1 = \int\limits_0^1\frac{xdx}{|\Delta_\pm(x)|}\frac{\Theta[t-x]}{\sqrt{t^2-x^2}}\frac{1}{\sqrt{1+\zeta^2_\pm x^2}}, \tag{B17}$$

$$\mathcal{I}^{\pm}_2 = \int\limits_0^1\frac{xdx}{|\Delta_\pm(x)|}\frac{\Theta[t-x]}{\sqrt{t^2-x^2}}, \tag{B18}$$

$$\mathcal{I}^{\pm}_3 = \int\limits_0^1\frac{xdx}{|\Delta_\pm(x)|}\frac{\Theta\left[|u_\pm(x,t)|-x\right]}{\sqrt{u^2_\pm(x,t)-x^2}}\frac{\mathrm{sgn}\left[u_\pm(x,t)\right]}{\sqrt{1+\zeta^2_\pm x^2}}. \tag{B19}$$

The terms $\mathcal{F}^{\pm\pm}_{\parallel,z}$, $\mathcal{F}^{\pm\mp}_{\parallel,z}$ listed in the Appendix A are reproduced after some straightforward calculations of $\mathcal{I}^{\pm}_{1,2,3}$ given above. Let us only note the complex range of integration in $\mathcal{I}^{\pm}_3$, which leads to a double spike structure of the the interband response functions. This feature reflects the presence of two nesting vectors of the Fermi surface at the interband transitions.

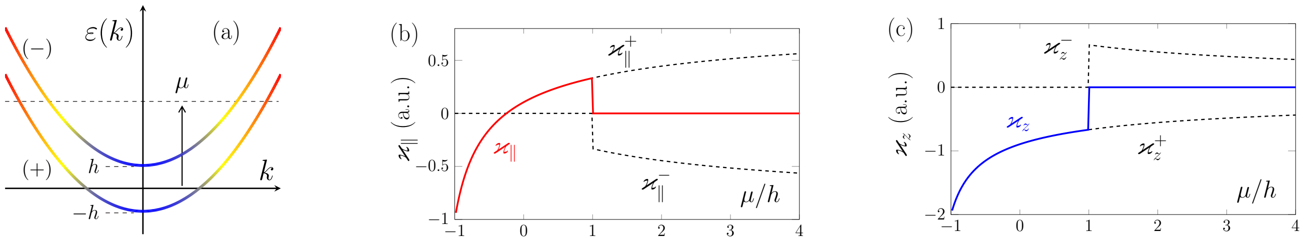

FIG. 1: (a) Parabolic-like electron spectrum Eq. 6 from main text, (b,c) the dependence of $\varkappa_{\parallel,z}$ on μ for $\xi = 0.5$.

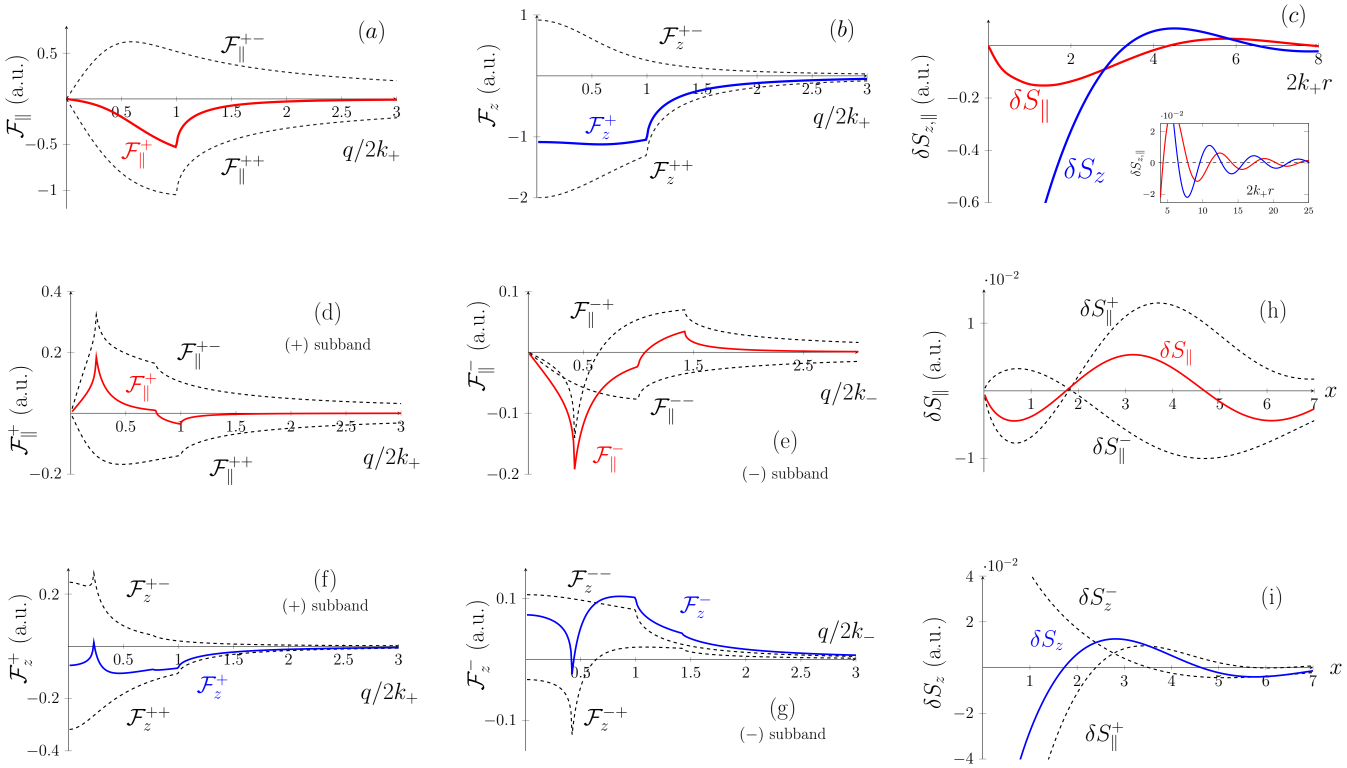

FIG. 2: (a,b,c) The dependence of $\mathcal{F}^+_{z,\parallel}$ on $q/2k_+$, and $\delta S_{z,\parallel}$ on $2k_+r$ in case of one filled spin subband ($\xi = 0.5$, $\mu = -0.4h$, $\zeta_+ = 1$), (d-g) the dependence of $\mathcal{F}^\pm_{z,\parallel}$ on $q/2k_\pm$, (h,i) the dependence of $\delta S_{z,\parallel}$ on $x = 2(\sqrt{2m\mu})r$ in case of two filled spin subbands, the parameters are $\mu = 3.5h$, $\xi = 0.5$, $\zeta_+ = 2.5$, $\zeta_- = 1.4$.

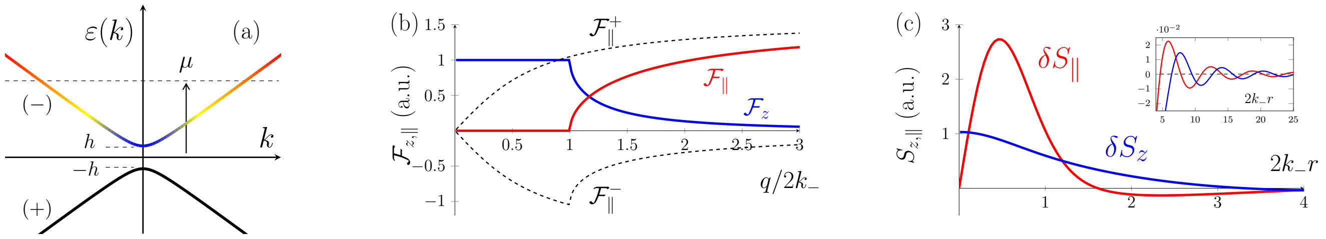

FIG. 3: (a) Dirac electron spectrum, (b) the dependence of $\mathcal{F}^\pm_{z,\parallel}$ on $q/2k_-$, (c) the dependence of $\delta S_{\parallel,z}$ on $2k_-r$, the inset shows the Friedel's oscillations. The parameters are $\mu = 2h$, $\zeta_- = 1.7$, $ak_- = 0.3$.
